# Preoperative TNF-α predicts uneventful postoperative outcomes in patients undergoing colorectal cancer surgery

**DOI:** 10.1038/s41598-025-06667-6

**Published:** 2025-07-01

**Authors:** Kornelija Rauduvytė, Paulina Kazlauskaitė, Marius Kryžauskas, Povilas Ignatavičius, Tomas Poškus, Rasa Sabaliauskaitė, Agata Mlynska, Agnė Šeštokaitė, Martynas Lukšta, Rimantas Baušys, Matas Jakubauskas, Augustinas Baušys

**Affiliations:** 1 Laboratory of Experimental Surgery and Oncology, Faculty of Medicine, Translational Health Research Institute, Vilnius, 08406 Lithuania; 2 Clinic of Gastroenterology, Nephrourology and Surgery, Institute of Clinical Medicine, Faculty of Medicine, Vilnius University, Vilnius, 03101 Lithuania; 3 Laboratory of Surgical Gastroenterology, Institute for Digestive Research, Medical Academy, Lithuanian University of Health Sciences, Kaunas, 44307 Lithuania; 4 Laboratory of Genetic Diagnostics, National Cancer Institute, Vilnius, 08406 Lithuania; 5 Laboratory of Immunology, National Cancer Institute, Vilnius, 08406 Lithuania; 6 Department of Chemistry and Bioengineering, Vilnius Gediminas Technical University, Vilnius, 10223 Lithuania

**Keywords:** Colorectal cancer, Postoperative complications, TNF-α, Uneventful course, Gastrointestinal cancer, Biomarkers

## Abstract

The development of novel biomarkers to predict postoperative complications (POCs) after colorectal cancer (CRC) surgery is essential, both for improving treatment outcomes and for facilitating the widespread use of same-day discharge following colectomy. This longitudinal observational study, a sub-study of a previous RCT, investigated the prognostic potential of a panel of novel biomarkers, including inflammatory cytokines (TNF-α, IL-1β, IL-2, IL-4, IL-6, IL-8, IL-10, IL-12p70, IL-17 A, INF-γ, IP-10, MCP-1, TGF-β1), alongside conventional biomarkers such as CRP and WBC levels, measured preoperatively and on postoperative day 6, for predicting POCs following CRC resection. Among 40 recruited patients, 38 were included in the final analysis. Patients who did not experience POCs had a significantly lower preoperative level of TNF-α (2.28 pg/mL vs. 68.77 pg/mL, *p* = 0.022). ROC curve analysis showed that TNF-α has significant (*p* = 0.012) discriminative capacity to distinguish between patients with POCs and those with an uneventful postoperative course, with an AUC of 0.722 (95% CI: 0.555, 0.889). Multivariable analysis suggested that a low preoperative TNF-α level (< 55.35 pg/mL) may be an independent predictor of an uneventful postoperative course (Odds Ratio = 0.137, 95% CI: 0.023, 0.836). This study showed that preoperative levels of TNF-α demonstrates a moderate discriminative capacity to distinguish between patients with POCs and those with an uneventful postoperative course in patients undergoing surgery for left-side CRC, although the findings are limited by the small sample size and should be validated in larger cohorts.

**Clinical Trial Registration:** NCT04013841, date of registration 2019-07-10.

## Introduction

Colorectal cancer (CRC) is the third most common malignancy and the second leading cause of cancer-related deaths worldwide. In 2022, CRC accounted for 1.9 million new cases (9.6%) and 904,000 deaths (9.3%)^1^. Surgery remains the primary treatment option for CRC.

Despite advancements in medical care, postoperative complications (POCs) persist in at least one-third of CRC patients^2,3^. These complications (e.g., infections^4^, anastomotic leakage^5^, etc.) increase in-hospital mortality risk, healthcare costs, impose significant financial burdens on patients, diminish quality of life, and hinder beneficial practices like same-day discharge (SDD) following colectomy^6–8^. SDD is an emerging protocol for selected low-risk CRC surgery patients, typically implemented within the broader framework of Enhanced Recovery After Surgery (ERAS) guidelines^9^.

Currently, there are no reliable preoperative tools or methods to accurately predict which patients are at risk of POCs. Identifying optimal biomarkers to forecast POCs could enable SDD to become the standard pathway for patients deemed unlikely to experience POCs. Moreover, early detection of POCs has been shown to improve outcomes in patients who do develop complications^10^. The most commonly utilized biomarkers for early detection of POCs include white blood cells count (WBC), procalcitonin, and C-reactive protein (CRP) levels^11–15^. While these biomarkers provide useful insights, they are typically measured in the postoperative setting and are aimed at early POCs detection only after complications have begun. To address this limitation, inflammatory cytokines – key regulators of the inflammatory response that stimulate CRP production^16,17^ – are being explored as promising non-invasive biomarkers for earlier detection of POCs^18^. One example of such cytokines is tumor necrosis factor-alpha (TNF-α), which has been shown to be elevated in advanced-stage CRC patients^19,20^. This underscores the growing recognition of inflammation and cytokine dysregulation as integral components of cancer initiation and progression, particularly in the context of CRC^21^.

To our knowledge, only one previous study has evaluated the prognostic role of preoperative TNF-α levels^22^. In contrast, our prospective study evaluated a broader panel of biomarkers, including those measured on postoperative day (POD) 6. Additionally, TNF-α can increase the production of other inflammatory cytokines, such as (interleukin) (IL)-8 and IL-6 in CRC patients^23^, which have already been proposed as potential prognostic indicators of POCs following colorectal surgery^24^. However, the lack of clinical evidence prevents TNF-α and other cytokines widespread adoption. Therefore, the aim of this study was to investigate the predictive value of inflammatory cytokines and conventional biomarkers, including CRP and WBC levels measured preoperatively, for identifying the risk of POCs following CRC resections.

## Materials and methods

### Study design and ethics

This longitudinal observational study is a sub-study of a previously conducted two-arm randomized controlled trial (RCT) registered on ClinicalTrials.gov (NCT04013841, 2019-07-10). The results of the RCT have been published previously^25^. Ethical approval for the study protocol was granted by the Vilnius Regional Bioethics Committee (25 June 2019; No. 2019/6˗1133˗631). The study was conducted in accordance with the principles outlined in the Declaration of Helsinki. All participants provided written informed consent prior to their involvement in the study.

## Study setting and participants

This study included patients aged 18 years or older who were scheduled to undergo elective left-sided colorectal resection for CRC between April and November 2021 at the National Cancer Institute in Vilnius, Lithuania. The exclusion criteria were as follows: (1) anticipation of an ileostomy; (2) allergy to oral preparation agents; (3) requirement for multivisceral surgery; (4) need for emergency surgery; (5) history of inflammatory bowel disease; (6) history of surgery affecting the gastrointestinal tract integrity; (7) clinical signs of a tumor obstructing the bowel lumen; (8) pregnancy. The POCs were defined by two oncologic surgeons based on comprehensive assessment that included laboratory results, radiological examinations, microbiological cultures or intraoperative findings in cases where reoperation was necessary. Based on the development of POCs, patients were categorized into two groups: the POC + group, consisting of patients who experienced POCs, and the POC- group, comprising patients who did not experience POCs. POCs were considered positive if the Clavien Dindo grading score was ≥ 1. Except for POCs all patients were treated according to the same postoperative treatment protocol.

## Study outcomes

This study evaluated the prognostic potential of a panel of novel biomarkers, including cytokines (TNF-α, IL-1β, IL-2, IL-4, IL-6, IL-8, IL-10, IL-12p70, IL-17 A, interferon gamma (INF-γ), interferon gamma-induced protein 10 (IP-10), monocyte chemoattractant protein-1 (MCP-1), and transforming growth factor beta (TGF-β1)), alongside conventional biomarkers such as CRP and WBC levels. The ability of these biomarkers to distinguish between patients with and without POCs following CRC resections was assessed using receiver operating characteristic (ROC) analysis and their corresponding area under the curve (AUC) values.

## Blood sample collection

Fresh blood samples were collected preoperatively (baseline) and on POD6. The samples were centrifuged at 3000 rpm for 10 min. at 4 °C. The serum was then collected in 1.5 mL tubes and immediately stored at -80 °C until further processing.

## Measurement of inflammatory biomarkers

The concentrations (pg/mL) of serum cytokines (TNF-α, IL-1β, IL-2, IL-4, IL-6, IL-8, IL-10, IL-12p70, IL-17 A, INF-γ, IP-10, MCP-1 and TGF-β1) were measured using the multiplex cytometric bead-based assay LEGENDplex™ Human Essential Immune Response Panel (13-plex) (Cat. No: 740930, Lot: B335764, BioLegend, San Diego, CA, USA) by the personnel blinded to the patient outcomes. The assay was conducted in accordance with the manufacturer’s instructions. The samples were analyzed with BS LSR II (BD Biosciences, Franklin Lakes, NJ, USA) flow cytometer. Analyte concentrations were determined using LEGENDplex™ Data Analysis Software (v2023-02-12, 58444) (BioLegend, USA). The lower detection limit of the assay for cytokines were: TNF-α 2.28 pg/mL, IL-1β 1.06 pg/mL, IL-2 0.942 pg/mL, IL-4 24.40 pg/mL, IL-6 0.717 pg/mL, IL-8 1.29 pg/mL, IL-10 0.52 pg/mL, IL-12p70 0.72 pg/mL, IL-17 A 1.34 pg/mL, INF-γ 1.933 pg/mL, IP-10 1.12 pg/mL, MCP-1 5.053 pg/mL and TGF-β1 13.85 pg/mL. Several measurements, including the median values for some cytokines, were at or near this threshold. When a value was at the detection limit, the lowest value of the detection range was imputed for inclusion in the final analysis. All samples for each cytokine were successfully examined and included in the final analysis. CRP (mg/L) and WBC (x10^9^/L) count were measured according to standard protocols in a routine clinical laboratory.

### Statistical analysis

Statistical analyses were performed using SPSS version 29.0.2.0 (IBM, Armonk, NY, USA) and R version 2024.09.1 (Integrated Development for R; RStudio, PBC, Boston, MA, USA). Data visualization was performed using GraphPad Prism 10.4.0 (Boston, MA, USA). Data normality was assessed using the Shapiro–Wilk test. Categorical variables were analyzed using the Fisher’s exact test or χ2 test, while continuous variables were compared using the Mann-Whitney U test. Continuous data were reported as medians with interquartile ranges (first quartile (Q1) to third quartile (Q3)).

ROC curve analysis was conducted using the “pROC” package to evaluate the sensitivity, specificity, positive predictive value (PPV), negative predictive value (NPV), and accuracy of each biomarker. ROC curves with *p* values ≤ 0.1 were plotted. Biomarker concentrations were categorized as high and low based on the best cut-off values determined by R using Youden’s index. Concentrations above the best cut-off value were classified as high, while those below were classified as low.

Univariate analysis was conducted to identify independent variables associated with an uneventful postoperative course, including all potential risk factors. Potential variables with a *p* value ≤ 0.150 in the univariate analysis were subsequently included in multivariable logistic regression models for further evaluation. Statistical significance was defined as *p* ≤ 0.05.

This study is a sub-study of a previously conducted RCT, all power calculations and sample size justification are provided in the original RCT and no formal sample size calculations were performed for this sub-study^25^.

## Results

### Baseline characteristics

**Fig. 1:**
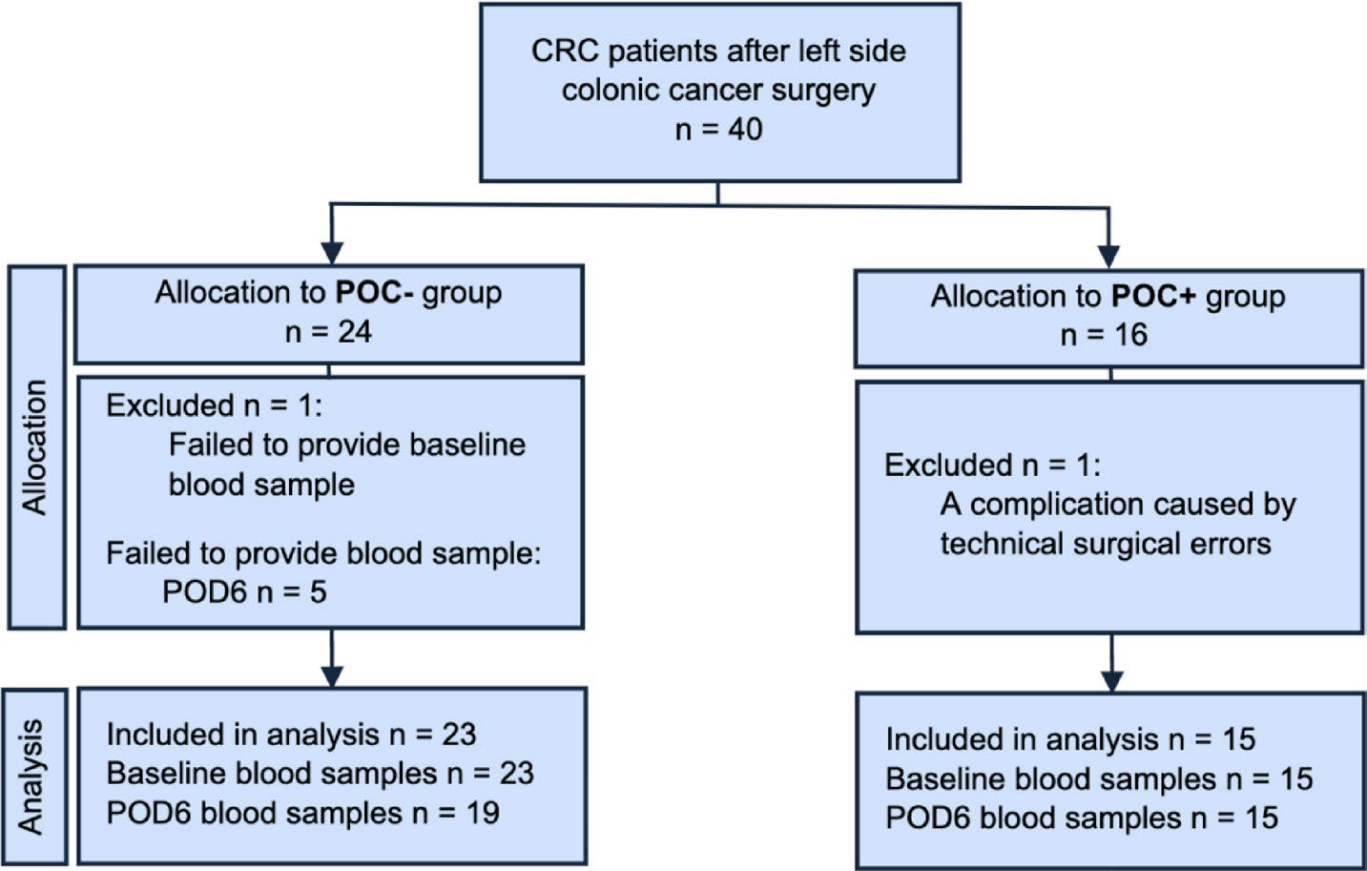
Flowchart of the analysis.

A total of 40 participants were recruited into the study (Fig. 1). In total two (5%) patients were excluded from further analysis (one from each group) for the following reasons: (1) one patient failed to provide preoperative blood samples (n = 1) and (2) one patient with a POC (n = 1) due to iatrogenic injury. After exclusion, 38 patients were included in the final analysis. Among them, 15 patients were allocated to the POC+ group and 23 patients to the POC- group. In the POC+ group, 6 out of 15 patients (40%) developed wound suppuration, 3 out of 15 (20%) experienced urinary tract infections, 2 out of 15 (13%) had intra-abdominal abscesses, 2 out of 15 (13%) suffered from postoperative ileus, 1 out of 15 (7%) experienced anastomotic leakage, and 1 out of 15 (7%) had other types of complications.

The POC- and POC+ groups were comparable in demographic, clinicopathological, and treatment characteristics (Table 1).

**Table 1:** Baseline and treatment characteristics.

Baseline characteristics		POC- (*n* = 23)	POC+ (*n* = 15)	*p* value
Age, years, median (Q1-Q3)		67 (53–70)	72 (59–77)	0.145
Gender, n (%)	Male	8 (34.8)	9 (60.0)	0.185
	Female	15 (65.2)	6 (40.0)	
Smoking status, n (%)	Smokers / ex-smokers	2 (8.7)	1 (6.7)	0.999
	Non-smokers	21 (91.3)	14 (93.3)	
Alcohol consumption, n (%)	Drinkers	11 (47.8)	7 (46.7)	0.999
	Non-drinkers	12 (52.2)	8 (53.3)	
Bowel preparation, n (%)	Oral preparation	12 (52.2)	7 (46.7)	0.999
	Rectal enema	11 (47.8)	8 (53.3)	
pT, n (%)	T0-2	8 (34.7)	2 (13.3)	0.251
	T3-4	13 (56.5)	12 (80.0)	
	Not applicable	2 (8.7)	1 (6.7)	
pN, n (%)	N0	15 (65.2)	7 (46.7)	0.288
	N1-2	6 (26.1)	7 (46.7)	
	Not applicable	2 (8.7)	1 (6.7)	
pM, n (%)	M0	21 (91.3)	12 (80.0)	0.153
	M1	0 (0.0)	2 (13.3)	
	Not applicable	2 (8.7)	1 (6.7)	
Clinical stage, n (%)	1	10 (43.5)	4 (26.7)	0.444
	2	4 (17.4)	2 (13.3)	
	3	9 (39.1)	9 (60.0)	
Pathological stage, n (%)	I-II	15 (65.2)	6 (40.0)	0.232
	III-IV	6 (26.1)	8 (53.3)	
	Not applicable	2 (8.7)	1 (6.7)	
Surgical approach, n (%)	Laparoscopic	18 (78.3)	9 (60.0)	0.285
	Open	5 (21.7)	6 (40.0)	
ASA, n (%)	1–2	21 (91.3)	11 (73.3)	0.188
	> 2	2 (8.7)	4 (26.7)	
CCI, n (%)	1–5	20 (87.0)	9 (60.0)	0.115
	> 5	3 (13.0)	6 (40.0)	
Length of surgery, minutes, median (Q1-Q3)		115 (95–150)	145 (100–155)	0.095

pT – pathological tumor stage; pN – pathological nodal stage; pM – pathological distant metastasis according to TNM classification; ASA – American Society of Anesthesiologists physical status; CCI – Charlson Comorbidity Index.

### Pre- and Post-operative differences of inflammatory biomarkers

The POC- group had significantly lower preoperative levels of serum TNF-α (2.28 pg/mL vs. 68.77 pg/mL, *p* = 0.022) (Table 2). Additionally, CRP and IP-10 were significantly lower in the POC- group on POD6 (*p* < 0.001 and *p* = 0.027, respectively). Other biomarkers did not show significant differences between the groups at any of the timepoints.

**Table 2:** The level of inflammatory biomarkers in the POC- and POC+ groups at different timepoints.

		POC- (*n* = 23)	POC+ (*n* = 15)	*p* value
WBC (x10^9^/L), median (Q1-Q3)	Baseline	6.90 (5.50–8.80)	6.98 (6.32–8.48)	0.860
	POD6	6.91 (5.56–8.56)	8.57 (6.05–9.76)	0.225
CRP (mg/L), median (Q1-Q3)	Baseline	2.70 (1.70–4.90)	4.40 (1.15–26.90)	0.426
	POD6	18.85 (12.48–36.30)	82.80 (56.00-137.40)	**< 0.001** ^*^
TNF-α (pg/mL), median (Q1-Q3)	Baseline	2.28 (2.28–19.51)	68.77 (2.28-594.84)	**0.022** ^*^
	POD6	2.28 (2.28–22.59)	2.28 (2.28-550.44)	0.732
IL-1β (pg/mL), median (Q1-Q3)	Baseline	54.20 (1.06-107.32)	5.77 (1.06-110.56)	0.359
	POD6	71.57 (2.68–179.80)	2.03 (1.06–84.48)	0.071
IL-2 (pg/mL), median (Q1-Q3)	Baseline	0.94 (0.94–9.73)	2.17 (0.94-20.00)	0.658
	POD6	1.95 (0.94-20.00)	2.28 (0.94–8.84)	0.784
IL-4 (pg/mL), median (Q1; Q3)	Baseline	24.40 (24.40-29.71)	24.40 (24.40-67.96)	0.813
	POD6	29.71 (24.40-66.35)	24.40 (24.40-195.37)	0.891
IL-6 (pg/mL), median (Q1-Q3)	Baseline	9.17 (2.22–31.28)	24.28 (8.61–62.57)	0.235
	POD6	35.85 (9.46-103.21)	103.21 (21.26-279.22)	0.128
IL-8 (pg/mL), median (Q1-Q3)	Baseline	7.15 (3.97–15.41)	15.81 (4.73–56.22)	0.101
	POD6	6.32 (3.34–51.04)	13.65 (4.62–44.63)	0.271
IL-10 (pg/mL), median (Q1-Q3)	Baseline	1.57 (0.87–3.75)	3.89 (1.57–5.63)	0.202
	POD6	3.19 (0.93–12.81)	4.25 (1.83–12.81)	0.537
IL-12p70 (pg/mL), median (Q1-Q3)	Baseline	0.72 (0.72–4.22)	1.17 (0.72–4.92)	0.273
	POD6	1.17 (0.72–7.41)	2.07 (0.72–17.19)	0.732
IL-17 A (pg/mL), median (Q1-Q3)	Baseline	2.64 (1.34–6.85)	5.26 (1.64–11.04)	0.391
	POD6	2.28 (1.34–15.86)	4.47 (1.34–28.97)	0.391
INF-γ (pg/mL), median (Q1-Q3)	Baseline	3.10 (1.93–22.63)	10.15 (1.93–78.26)	0.300
	POD6	3.73 (1.93–23.82)	8.70 (1.93–58.45)	0.973
IP-10 (pg/mL), median (Q1-Q3)	Baseline	12.46 (1.12–83.24)	52.03 (1.12-127.12)	0.359
	POD6	62.52 (1.12–88.62)	135.98 (10.66-222.03)	**0.027** ^*^
MCP-1 (pg/mL), median (Q1-Q3)	Baseline	679.11 (464.86-875.98)	455.65 (88.58-998.41)	0.235
	POD6	821.93 (288.66-1106.03)	464.57 (74.59-919.88)	0.190
TGF-β1 (pg/mL), median (Q1-Q3)	Baseline	139.71 (74.32-418.27)	148.41 (82.90-597.81)	0.953
	POD6	249.94 (13.85-539.67)	285.37 (148.41-465.35)	0.656

^*^ Statistically significant (*p* ≤ 0.05).

### Biomarkers for predicting an uneventful postoperative course

**Fig. 2:**
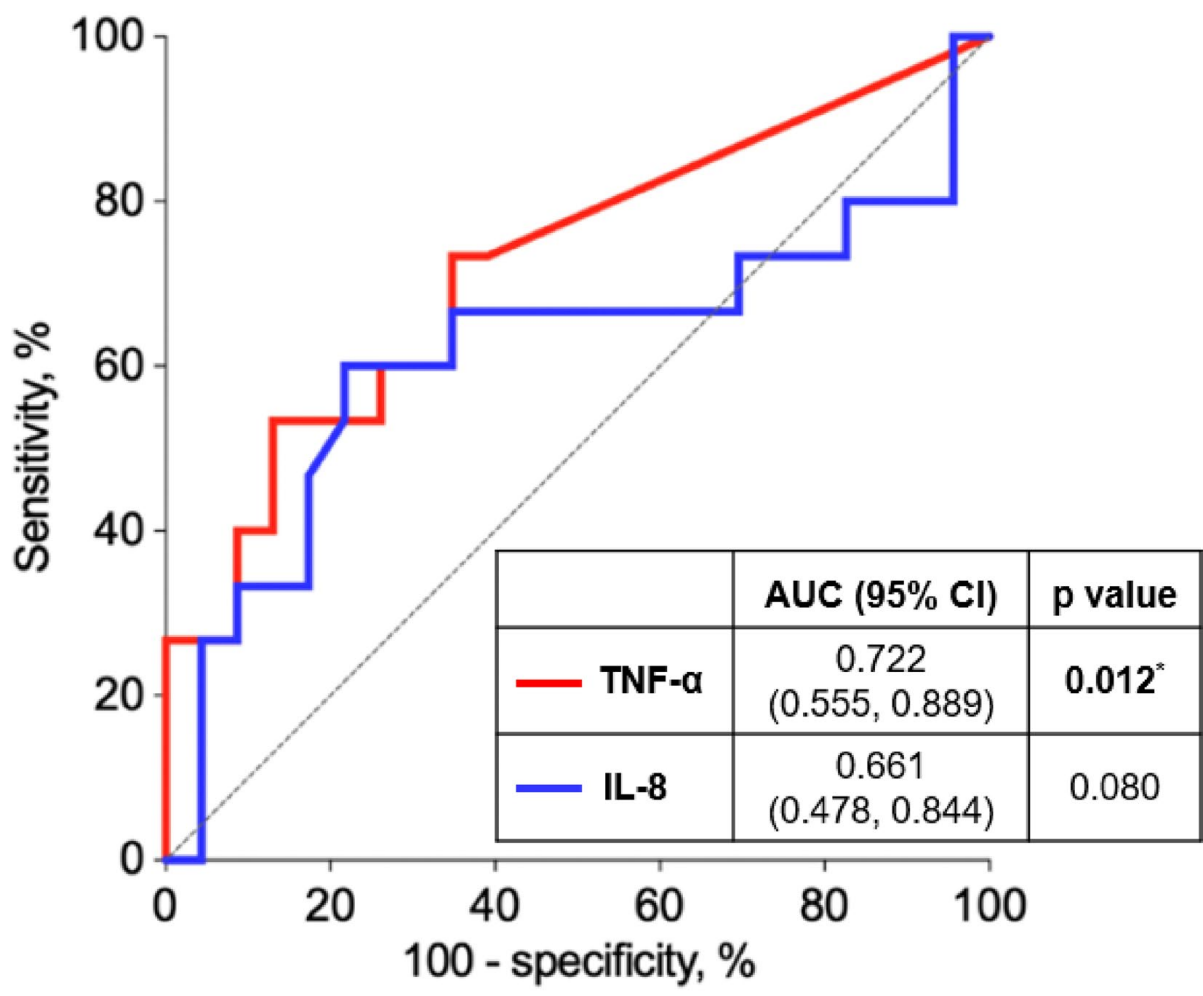
ROC curve analysis of preoperative TNF-α (red, AUC 0.722) and IL-8 (blue, AUC 0.661) to predict uneventful course. ^*^Statistically significant (*p* ≤ 0.05); AUC – area under the curve; CI – confidence interval.

ROC curve analysis of the preoperative biomarker level indicated that only TNF-α demonstrated a significant discriminative capacity to predict an uneventful postoperative course, with an AUC of 0.722 (95% CI: 0.555, 0.889). The sensitivity and specificity of TNF-α were 53% and 87%, respectively. IL-8 exhibited a notable trend to predict an uneventful postoperative course but did not reach statistical significance (Table 3; Fig. 2).

**Table 3:** ROC curve analysis of preoperative biomarkers for predicting an uneventful course.

	Cut-off value	AUC (95% CI)	Sensitivity	Specificity	PPV	NPV	Accuracy	Youden index	*p* value
WBC	6.18	0.517 (0.331, 0.704)	0.800	0.391	0.462	0.750	0.553	0.191	0.853
CRP	8.55	0.587 (0.356, 0.818)	0.462	0.947	0.857	0.720	0.750	0.409	0.448
TNF-α	55.35	0.722 (0.555, 0.889)	0.533	0.870	0.727	0.741	0.737	0.403	**0.012** ^*^
IL-1β	6.41	0.591 (0.396, 0.786)	0.600	0.739	0.600	0.739	0.684	0.339	0.355
IL-2	1.63	0.543 (0.363, 0.724)	0.600	0.565	0.474	0.684	0.579	0.165	0.650
IL-4	51.30	0.525 (0.362, 0.687)	0.333	0.826	0.556	0.655	0.632	0.159	0.801
IL-6	12.05	0.616 (0.423, 0.809)	0.667	0.652	0.556	0.750	0.658	0.319	0.232
IL-8	9.99	0.661 (0.478, 0.844)	0.667	0.696	0.588	0.762	0.684	0.363	**0.080**
IL-10	3.82	0.625 (0.434, 0.816)	0.533	0.783	0.615	0.720	0.684	0.316	0.193
IL-12p70	0.75	0.609 (0.430, 0.788)	0.667	0.565	0.500	0.722	0.605	0.232	0.249
IL-17 A	3.78	0.586 (0.396, 0.775)	0.600	0.609	0.500	0.700	0.605	0.209	0.372
INF-γ	7.81	0.601 (0.411, 0.792)	0.600	0.696	0.563	0.727	0.658	0.296	0.300
IP-10	88.57	0.590 (0.401, 0.779)	0.467	0.826	0.636	0.704	0.684	0.293	0.352
MCP-1	463.91	0.616 (0.407, 0.825)	0.600	0.783	0.643	0.750	0.711	0.383	0.266
TGF-β1	572.49	0.506 (0.308, 0.704)	0.267	0.870	0.571	0.645	0.632	0.137	0.954

^*^Statistically significant (*p* ≤ 0.05); AUC – area under the curve; CI – confidence interval; PPV – positive predictive value; NPV – negative predictive value.

### Multivariable analysis: risk factors associated with an uneventful postoperative course

Multivariable regression analysis identified risk factors associated with an uneventful postoperative course following left-sided CRC surgery (Table 4). The analysis suggested that only a low preoperative level of TNF-α (< 55.35 pg/mL) may be an independent predictor of an uneventful postoperative course (Odds Ratio = 0.137, 95% CI: 0.023, 0.836). Further, the clinicopathologic and treatment characteristics of patients with a low and high TNF-α levels were not different, except significantly higher proportion of patients in high TNF-α group had higher ASA score (Table S1).

**Table 4:** Multivariable analysis of preoperative risk factors associated with an uneventful postoperative course.

Risk factor		Odds ratio	95% CI	*p* value
TNF-α	< 55.35 pg/mL	0.137	0.023, 0.836	**0.031** ^*^
IL-8	< 9.99 pg/mL	0.302	0.063, 1.447	0.134
CCI	< 5	0.214	0.022, 2.037	0.180
Age		0.973	0.890, 1.064	0.548

^*^Statistically significant (*p* ≤ 0.05); CCI – Charlson Comorbidity Index; CI – confidence interval.

## Discussion

This study investigated a panel of inflammatory cytokines alongside conventional biomarkers such as CRP and WBC levels as potential novel biomarkers for predicting POCs in CRC patients. The main findings reveal that higher baseline levels of TNF-α are associated with CRC patients who develop POCs. Furthermore, TNF-α showed a notable discriminative ability to differentiate between patients with POCs and those with an uneventful postoperative course, achieving an AUC of 0.722 (95% CI: 0.555, 0.889), with a sensitivity of 53% and specificity of 87%, indicating an 87% probability of accurately identifying patients without POCs.

Interest in biomarkers capable of predicting POCs as early as possible has been growing in recent years. Accurate preoperative risk assessment can enhance hospital resources utilization^26^ and improve surgical decision-making^27^, including guiding SDD for CRC surgery protocol. Serum concentrations of pro- or anti-inflammatory biomarkers have shown promise as potential predictors of POC following colorectal surgery^18^. Cytokines such as TNF-α and IL-1 play pivotal roles in initiating and regulating the inflammatory response after acute tissue injury caused by surgery. These cytokines are predominantly released by activated macrophages and monocytes and serve as potent inducers of other inflammatory mediators, such as IL-6^28,29^. IL-6, in turn, drives the production of CRP during the acute phase of inflammation^16^, linking these biomarkers to the postoperative inflammatory cascade. Currently, CRP remains the gold standard biomarker for detecting POCs, including infectious complications^15^ such as anastomotic leakage^30^. However, its predictive value peaks relatively late, typically by POD5, limiting its utility for early intervention^15^. In this study, CRP demonstrated its value by showing elevated levels in patients with POCs on POD6. However, preoperative CRP levels lacked any significant predictive capacity, underscoring the need for biomarkers that can provide earlier risk stratification. This suggests that while CRP is effective for monitoring POC at later timepoints, earlier released biomarkers like IL-6 and TNF-α, which rise rapidly after acute phase injury and induce CRP elevation, may provide better preoperative risk assessment and early detection of POC.

Considering previously mentioned inflammatory response cascade triggered by surgical injury, TNF-α emerges as a plausible biomarker for early prediction of POCs. As a key regulator of the inflammatory response, TNF-α plays a critical role not only in initiating cytokine release, but also in promoting cell migration, and influencing metastatic phenotypes^31^. Its involvement in cancer progression further underscores its significance in the context of CRC^32^. These characteristics suggest the hypothesis that TNF-α peaks earlier than other biomarkers, making it a potential candidate for preemptive risk assessment.

From a clinical perspective, the preoperative measurement of TNF-α could serve as a valuable adjunct to existing risk stratification tools such as ASA classification or the Charlson Comorbidity Index. Rather than replacing these tools, TNF-α would provide complementary, dynamic insight into a patient’s inflammatory baseline – something that static scoring systems lack. This integration could significantly enhance the selection process for SDD protocols after colectomy, by enabling more accurate patient selection, thereby reducing medical risks and optimizing hospital resource utilization. This could lead to a more efficient, cost-effective, and personalized surgical pathway while simultaneously improving patient satisfaction.

To our best knowledge only one previous study investigated predictive potency of preoperative TNF-α levels. A study by Liu et al. examined serum TNF-α levels in 200 CRC patients undergoing primary tumor resection and found significantly higher preoperative TNF-α levels in those who developed specifically infectious POCs^22^. However, contrary to our study, the magnitude of the difference in TNF-α levels between patients with and without complications in their study was 1.6-fold (7.2 ng/L vs. 4.5 ng/L), which was considerably smaller than the 30.2-fold difference observed in our study (68.77 pg/mL vs. 2.28 pg/mL). The difference may be due to the methods used for TNF-α measurement, as Liu et al. used ELISA, while our study used a bead-based immunoassay^33^. Moreover, our study highlighted the moderate discriminative capacity of TNF-α to distinguish between patients with any type of POCs and those with an uneventful postoperative course. In the present study, we focused solely on serum TNF-α levels; however, its abundance in other locations, such as intraabdominal fluid, may also provide valuable clinical insights. Previous studies have shown that elevated levels of IL-6, IL-10, and TNF-α in the peritoneal drainage fluid as early as POD1 were associated with an increased risk of anastomotic leakage^34^. In contrary the study by Dimopoulou et al. found no significant differences in preoperative TNF-α levels between patients who developed complications and those who did not following elective major abdominal surgery^35^. This suggests that TNF-α might not universally predict POCs across all types of visceral surgeries. Therefore, further research is needed to determine whether TNF-α can serve as a universal biomarker for POCs in visceral surgeries or if its predictive value is more specific to CRC surgery.

Additionally, the clinicopathologic and treatment characteristics of patients with low and high preoperative TNF-α levels were comparable, suggesting that TNF-α levels may serve as an independent marker rather than simply reflecting underlying differences in baseline clinical factors, such as clinical stage. However, some studies found that high levels of TNF-α are found in advanced-stage CRC patients^19,20^. Furthermore, we observed a notable difference of patients with elevated TNF-α levels having a higher ASA score. This finding aligns with existing literature indicating that elevated TNF-α levels are associated with various inflammatory conditions and comorbidities^36^, whereas high ASA score is associated with higher mortality risk^37^ and POCs^38^. While this association supports the potential relevance of TNF-α in preoperative risk stratification, it also underscores the need to consider broader clinical context when interpreting cytokine profiles.

Our finding that patients who developed POCs had higher preoperative TNF-α levels, despite no associations with clinicopathologic variables (except ASA score, as discussed before), raises the possibility that elevated TNF-α may reflect a state of pre-existing immune activation. TNF-α is a key pro-inflammatory cytokine involved in immune system activation, leukocyte recruitment, and vascular permeability – mechanisms that can amplify the body’s inflammatory response to surgical stress^39^. At the molecular level, TNF-α is can trigger apoptosis via caspase-8 activation^40^ and impair mitochondrial function^41^, processes that are detrimental to tissue regeneration and barrier integrity^42–44^. One plausible explanation is that elevated TNF-α levels may indicate a pre-existing pro-inflammatory or immune primed state, potentially stemming from gut barrier dysfunction^45^ or microbial translocation^46^. This heightened immune reactivity could impair normal healing, promote tissue damage, and increase susceptibility to infections or other POCs. Understanding this underlying immune primed state may not only enhance the predictive value of TNF-α but also open avenues for targeted prehabilitation strategies aimed at mitigating postoperative risk.

Another biomarker that showed a tendency to predict postoperative outcomes in the present study was IL-8, although it did not reach statistical significance. IL-8 has been demonstrated to be superior to CRP in predicting POCs, particularly in the context of liver surgery for CRC metastases^47^. Furthermore, TNF-α can stimulate the production of other pro-inflammatory cytokines, such as IL-8 in CRC^23^. Although IL-8 alone did not reach statistical significance in our study, it may still enhance predictive accuracy when used in combination with other markers. Biomarkers with moderate individual effects can provide additive or synergistic value in multivariate models, particularly when capturing complementary aspects of the inflammatory response. Given this connection, IL-8, alongside TNF-α, could be a promising candidate for inclusion in future combinatorial biomarker panels aimed at improving the preoperative prediction of POCs in CRC surgery as well, although its value has to be confirmed in future studies.

The present study has noteworthy limitations. First, the small cohort size limits the statistical power and generalizability of the findings. Second, this was a sub-study of a previous RCT that included only left-side CRC patients, so the findings may not be fully generalizable to the entire CRC cohort, particularly for patients with right-side tumors. Third, some cytokine measurements were below the detection limit, potentially introducing bias and skewing the distribution. The fourth drawback of the study is the timing of sample collection. After collecting preoperative samples, we only collected postoperative samples on POD6. This limited our ability to evaluate the biomarkers’ prognostic capacity in the early postoperative setting, particularly within the first few PODs. While our findings provide valuable insights, additional studies involving larger patient cohorts and patients with different tumor localizations within the colon are needed to confirm TNF-α and other cytokines as reliable preoperative biomarkers for discriminating between patients who will develop POCs and those with an uneventful postoperative course.

In summary, our findings suggest TNF-α is a promising biomarker for preoperative risk stratification. Identifying individuals with low TNF-α levels could support the safe implementation of SDD protocols, optimizing resources use without compromising patient outcomes. Conversely, patients with elevated TNF-α levels may benefit from intensified perioperative strategies, such as prolonged prehabilitation, enhanced infection prevention protocols, or alternative surgical planning (e.g., lower risk anastomoses). The 30-fold difference between groups indicates a strong discriminatory signal with clinical relevance. While current testing may limit bedside use, this magnitude supports the development of rapid assays. A key strength of this study is the preoperative measurement of TNF-α and its predictive value, independent of most clinicopathologic factors. As a next step, a multicenter validation study is needed to confirm these findings and assess the value of both pre- and postoperative cytokine profiling.

## Conclusions

This study showed that preoperative levels of TNF-α demonstrates a moderate discriminative capacity to distinguish between patients with POCs and those with an uneventful postoperative course in patients undergoing surgery for left-side CRC.

## Electronic supplementary material

Below is the link to the electronic supplementary material.

Supplementary Material 1

## Data Availability

All datasets used in this study are available from the corresponding author upon reasonable request.
