# Supplementary material for: Preoperative TNF-α predicts uneventful postoperative outcomes in patients undergoing colorectal cancer surgery
Source: Sci Rep. 2025 Jul 1;15:21878. doi: 10.1038/s41598-025-06667-6 (PMC12216408; doi:10.1038/s41598-025-06667-6)
Supplement: Supplementary file 1 — Supplementary Material 1 [file 41598_2025_6667_MOESM1_ESM.docx]

**Preoperative TNF-α Predicts Uneventful Postoperative Outcomes in Patients Undergoing Colorectal Cancer Surgery**

Kornelija Rauduvytė^1^, Paulina Kazlauskaitė^1^, Marius Kryžauskas^2, 3^, Povilas Ignatavičius^3^, Tomas Poškus^1^, Rasa Sabaliauskaitė^4^, Agata Mlynska^5, 6^, Agnė Šeštokaitė^4^, Martynas Lukšta^2^, Rimantas Baušys^1^, Matas Jakubauskas^1^, Augustinas Baušys^1, *^

^1^Laboratory of Experimental Surgery and Oncology, Translational Health Research Institute, Faculty of Medicine, 08406 Vilnius, Lithuania

^2^Clinic of Gastroenterology, Nephrourology and Surgery, Institute of Clinical Medicine, Faculty of Medicine, Vilnius University, 03101 Vilnius, Lithuania

^3^Laboratory of Surgical Gastroenterology, Institute for Digestive Research, Medical Academy, Lithuanian University of Health Sciences, 44307 Kaunas, Lithuania

^4^Laboratory of Genetic Diagnostics, National Cancer Institute, 08406 Vilnius, Lithuania

^5^Laboratory of Immunology, National Cancer Institute, 08406 Vilnius, Lithuania

^6^Department of Chemistry and Bioengineering, Vilnius Gediminas Technical University, 10223 Vilnius, Lithuania

^*^ Corresponding author: Augustinas Baušys, Laboratory of Experimental Surgery and Oncology, Translational Health Research Institute, Faculty of Medicine, 03101 Vilnius, Lithuania (e-mail: augustinas.bausys@mf.vu.lt)

**Supplementary material**

**Table S1** Comparison between low and high TNF-α level groups

| **Baseline characteristics** | | **Low TNF-α**  **(<55.35 pg/mL)**  **(n = 27)** | **High TNF-α**  **(≥55.35 pg/mL)**  **(n = 11)** | **p value** |
| --- | --- | --- | --- | --- |
| **Age, years, median (Q1-Q3)** | | 67 (55-72) | 74 (62-85) | **0.035^*^** |
| **Gender, n (%)** | Male | 12 (44.4) | 5 (45.5) | 1.000 |
|  | Female | 15 (55.6) | 6 (54.5) |  |
| **Smoking status,**  **n (%)** | Smokers / ex-smokers | 3 (11.1) | 0 (0.0) | 0.542 |
|  | Non-smokers | 24 (88.9) | 11 (100.0) |  |
| **Alcohol consumption, n (%)** | Drinkers | 14 (51.9) | 4 (36.4) | 0.485 |
|  | Non-drinkers | 13 (48.1) | 7 (63.6) |  |
| **Bowel preparation,**  **n (%)** | Oral preparation | 13 (48.1) | 6 (54.5) | 1.000 |
|  | Rectal enema | 14 (51.9) | 5 (45.5) |  |
| **pT, n (%)** | T1-2 | 7 (25.9) | 2 (18.2) | 0.692 |
|  | T3-4 | 17 (63.0) | 8 (72.7) |  |
|  | Not applicable | 3 (11.1) | 1 (9.1) |  |
| **pN, n (%)** | N0 | 17 (63.0) | 5 (45.5) | 0.444 |
|  | N1-2 | 8 (29.6) | 5 (45.5) |  |
|  | Not applicable | 2 (7.4) | 1 (9.1) |  |
| **pM, n (%)** | M0 | 24 (88.9) | 9 (81.8) | 0.496 |
|  | M1 | 1 (3.7) | 1 (9.1) |  |
|  | Not applicable | 2 (7.4) | 1 (9.1) |  |
| **Clinical stage,**  **n (%)** | 1 | 10 (37.0) | 4 (36.4) | 0.430 |
|  | 2 | 3 (11.1) | 3 (27.3) |  |
|  | 3 | 14 (51.9) | 4 (36.4) |  |
| **Pathological stage,**  **n (%)** | I-II | 16 (59.3) | 5 (45.5) | 0.737 |
|  | III-IV | 9 (33.3) | 5 (45.5) |  |
|  | Not applicable | 2 (7.4) | 1 (9.1) |  |
| **Surgical approach,**  **n (%)** | Laparoscopic | 18 (66.7) | 9 (81.8) | 0.452 |
|  | Open | 9 (33.3) | 2 (18.2) |  |
| **ASA, n (%)** | 1-2 | 25 (92.6) | 7 (63.6) | **0.047^*^** |
|  | >2 | 2 (7.4) | 4 (36.4) |  |
| **CCI, n (%)** | 1-5 | 22 (81.5) | 7 (63.6) | 0.401 |
|  | >5 | 5 (18.5) | 4 (36.4) |  |
| **Length of surgery, minutes,**  **median (Q1-Q3)** | | 115 (100-150) | 150 (140-155) | 0.071 |

^*^Statistically significant (p ≤ 0.05); pT – pathological tumor stage; pN – pathological nodal stage; pM – pathological distant metastasis according to TNM classification; ASA – American Society of Anesthesiologists physical status; CCI – Charlson Comorbidity Index
